# It Was Not the Perfect Storm: The Social History of the HIV-2 Virus in Guinea-Bissau

**DOI:** 10.3390/tropicalmed8050261

**Published:** 2023-05-02

**Authors:** Jorge Varanda, José Maurício Santos

**Affiliations:** 1 Centre for Research in Anthropology (CRIA-UC), Department of Life Sciences, University of Coimbra, 3000-456 Coimbra, Portugal; 2 Global Health and Tropical Medicine, Institute of Hygiene and Tropical Medicine-NOVA-Lisbon (GHTM-UNL), Rua da Junqueira, 100, 1349-008 Lisboa, Portugal; 3 Centre for Geographical Studies, Institute of Geography and Spatial Planning, Universidade de Lisboa, 1600-276 Lisboa, Portugal; 4 Associated Laboratory TERRA, 1349-017 Lisboa, Portugal

**Keywords:** HIV-2, disease emergence, West Africa, zoonosis, biomedical technology, historical epidemiology

## Abstract

The perfect storm model that was elaborated for the HIV-1M pandemic has also been used to explain the emergence of HIV-2, a second human immunodeficiency virus-acquired immunodeficiency syndrome (HIV-AIDS) that became an epidemic in Guinea-Bissau, West Africa. The use of this model creates epidemiological generalizations, ecological oversimplifications and historical misunderstandings as its assumptions—an urban center with explosive population growth, a high level of commercial sex and a surge in STDs, a network of mechanical transport and country-wide, en masse mobile campaigns—are absent from the historical record. This model fails to explain how the HIV-2 epidemic actually came about. This is the first study to conduct an exhaustive examination of sociohistorical contextual developments and align them with environmental, virological and epidemiological data. The interdisciplinary dialogue indicates that the emergence of the HIV-2 epidemic piggybacked on local sociopolitical transformations. The war’s indirect effects on ecological relations, mobility and sociability were acute in rural areas and are a key to the HIV-2 epidemic. This setting had the natural host of the virus, the population numbers, the mobility trends and the use of technology on a scale needed to foster viral adaptation and amplification. The present analysis suggests new reflections on the processes of zoonotic spillovers and disease emergence.

## 1. Introduction

The perfect storm model elaborated for the HIV-1M pandemic is used to explain the emergence of HIV-2, a second human immunodeficiency virus-acquired immunodeficiency syndrome (HIV-AIDS) that became epidemic in Guinea-Bissau (GB), West Africa [1,2,3,4]. Unfortunately, such mimicking has its flaws. The occurrence of a perfect storm requires the following conditions—an urban center with explosive population growth, commercial sex leading to a surge in STDs and amplification via iatrogenic routes present in *en masse* mobile campaigns [5,6]. Our work shows that none of these conditions were present in Guinea-Bissau.

The discovery of HIV-2 in Senegal in 1985 [7], later confirmed in Paris in two hospitalized patients from Guinea-Bissau and Cape Verde [8], prompted worldwide surprise. However, it was the unexpectedly high prevalence of HIV-2 in Guinea-Bissau (5–12%), compared to neighboring countries (1%) [9], that led to the country’s designation as the epicenter [10]. This was an unlikely setting for an unlikely HIV-AIDS epidemic [11]: How did this virus become an epidemic? According to the dominant narrative, the HIV-2 epidemic occurred after the virus traveled thousands of kilometers from the Ivory Coast into a hypothetical densely populated major urban center—Bissau—where it found fertile ground in which to spread due to commercial sex and a high prevalence of sexually transmitted diseases (STDs). It then expanded throughout the country via returning laborers and anti-sleeping sickness and tuberculosis campaigns, that were active throughout the liberation/colonial war, and through traditional cultural practices (excision and circumcision) [1,2,3,10,12,13,14,15,16,17,18,19]. We argue that mimicking the model to HIV-2 in Guinea-Bissau is based on apparent similarities and not evidence. This results in epidemiological generalizations, ecological oversimplifications and historical misunderstandings. The model fails to explain why Guinea-Bissau became an epicenter despite being a rural country with no urban centers or modern transport, and a weak public health system struggling to stay afloat amidst a colonial/liberation armed conflict. It does not explain how the tiny village of Caió, on the country’s west coast, presented the highest prevalence in the world in 1987 with 7.9 percent [2,10]. In a nutshell: What made Guinea-Bissau exceptional, given that it does not comply with the model’s own ‘rules’?

By asserting the existence of a single road to Rome, or from animal virus to epidemic, the perfect storm molds the ‘real’ to fit its own narrative. This translates into tacitly fostering ‘the urban’ as the ignitor while silencing the rural environment. The comprehensive interdisciplinary dialogue led to a social history of biomedical care and historical epidemiology of 20th-century Guinea-Bissau which, in turn, allows us to move past the perfect storm narrative with its one-size-fits-all theory [20,21].

The historical record reveals that the imposition of colonial rule was a long conflictual process in which biomedical care played an important role [22,23]. While the military pacification campaigns subjugated the population, it would take several decades for the establishment of the colonial administration throughout the territory. The weak agricultural economy and scant colonial investment emphasized the rurality of the country. The liberation war (1963–1974) was a particular time that contributed to unusual changes in population mobility, sociability and ecological relations in the hinterland. Surprisingly, Portuguese public health actions increased during the conflict, that is, the public health approach that further favored injection procedures. The result was a surge in injections in rural areas, which provided the bridge for transferring a new virus.

The spillover and emergence of HIV-2 have been seen as unexpected and unlikely by many authors because these assumptions enforce conformation to the experience of HIV-1M. The perfect storm model is detrimental to both the understanding of viral emergence and the elaboration of new preparedness plans. It fails to acknowledge other emergence pathways, timing, contexts or factors. The careful integration of socio–historical–political and ecological developments with virological and epidemiological data, aligned with an integrative One Health *gestalt*, produces a new robust analysis of disease emergence and more effective public health policies.

### Beyond One-Size-Fits-All—Re-Centering on the Rural Landscape

Like its main counterpart, HIV1-M, HIV-2 adapted from the simian immunodeficiency (SIV) virus [18]. However, it originated from a virus that has been present in small old-world monkeys (*Cercocebus atys atys*, the sooty mangabey, or grey monkey in Guinean *kriol*) for hundreds of thousands of years: SIVsmm [24,25,26]. The lack of research on SIVs in Guinea-Bissau and the integration of empirical historical research led to the acceptance of the perfect storm model. 

The existing phylogenetic data on HIV-2 raises more questions than answers. The putative origin of HIV-2 in Taï Forest, the Ivory Coast [27], in the 1920s–1950s [1938 (1928–1947) [2]/1940 (1924–56) [1]] is severely limited by a lack of evidence and dismisses local ecological possibilities. The mysterious arrival of the virus in Bissau was followed by an epidemic spread in 1955–1970 [1,2]. The existence of *Cercocebus atys atys* monkeys [28], and the high prevalence of HIV-2, in Guinea-Bissau corroborate recent arguments for an independent founding effect [29]. While the existence of nine HIV-2 strains [29,30] might indicate that SIVs ‘jump easily’ from sooty mangabeys, the absence of more global epidemics shows how hard they find it to both adapt and spread more widely among humans [31,32]. Attention to the biological differences between the HIVs is central to understanding the possibility of different emerging pathways. HIV-2 failed where HIV-1M succeeded: to be sexually transmissible and forego the need for a technological ride [29,33,34,35,36]. 

The jump and adaptation process might have occurred along the lines of the serial passage, which proposes that successive parenteral transmission of SIV in humans was the root cause of SIVsmm adaptation to humans [32,37]. This framework in turn calls two biomedical technologies as the main candidates for parenteral exposure: blood transfusions (90% replicating the viral load) and unsterile injections (0.3–0.6%) [36,38]. This reinforces the role of social factors in the success of interspecies viral jumps.

**Figure 1:**
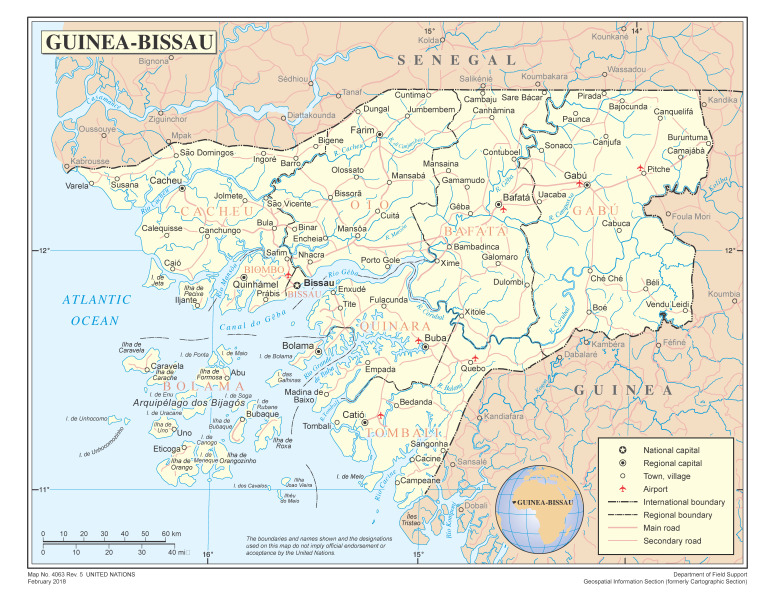
Map of Guinea-Bissau, https://www.un.org/geospatial/content/guinea-bissau (accessed on 26 March 2021).

Guinea-Bissau was a small rural country (Figure 1). Despite the small size, Portugal employed military pacification campaigns to occupy the land in the 1930s. Colonial census, local and administrative reports and colonial ethnographies provide a detailed image of the rural population and the absence of any network of cities, let alone a ‘major urban African city’ or railroad or mechanical transports. The existing urban areas were in fact small towns with no industry as the country’s economy rested on rice and nut crops. The bulk of the population lived in villages that dotted the hinterland; that is, it was a rural community. 

Changes in mobility and sociability across the colonial period favored a closer relationship between humans, non-human primates and biomedical technology, which points to the rural landscape as the key geography for this epidemic. The archival record strengthens this view, telling us that the urban landscape was tiny and that there were no underlying STD epidemics. 

Explaining the course of this (unexpected and unlikely) epidemic requires new kinds of evidence and analysis. The present article achieves this by producing a new narrative and presenting new data on locations, as well as a new timeframe and interplay of factors (biological socio-political and environmental), that explain the HIV-2 emergence. This case calls for a more detailed interdisciplinary analysis of zoonotic emergencies [21,39,40] and new ways of looking at the epidemic process [41,42,43,44,45].

## 2. Materials and Methods

New historical data and an in-depth interdisciplinary discussion are required to progress our understanding of the emergence of HIV. This paper is the result of extensive archival research and intense dialogue between different areas of knowledge, such as virology, biology, epidemiology and primatology and/or ecology, as well as history and anthropology. Research was conducted in 2015–2017 at several Portuguese archives: Arquivo Histórico Ultramarino (AHU), Arquivo Histórico Militar, Torre do Tombo National Archive (for the Portuguese Secret Police—Polícia Internacional e de Defesa do Estado, PIDE), *Centro de Intervenção para o Desenvolvimento Amílcar Cabral*), *Casa Comum*, which houses a vast body of material from the liberation movement (Partido Africano para a Independência da Guiné e Cabo Verde, PAIGC), and libraries such as the National Library. Wide-ranging primary data, such as colonial administration, public health and secret military reports and ethnographies and anthropological works covering 50 years, were considered for the first time. 

All qualitative data were analyzed for content and discourse and were coded. To ensure accuracy, the data were triangulated and the historical, social and cultural perspectives that led to the production of the different types of information were considered. This enabled us to establish a more robust hierarchy of credibility and ponder the possible bias. The results allow us to: elaborate on the geography of sooty mangabeys and the human population and its mobility; perform an epidemiological characterization of this period; detail the uses of biomedical technology by public health programs; and assess the dominance of ‘the rural’ in the country. 

## 3. Results

The historical record shows that the imposition of colonial rule in Guinea-Bissau was a slow, violent, fragmented process in which biomedical care was central. After 1963, the liberation/colonial war brought humans, monkeys and viruses closer together, and, in parallel with the surge in the use of (unsterile) injections, this facilitated the adaptation and spread of the virus.

### 3.1. The Hinterland and Its Transformations 

Guinea-Bissau presented no major urban center or network of cities during the colonial period. By 1960, the new colonial capital, Bissau, had some 25,000 inhabitants and no real sex-ratio imbalance (Table 1). In reality, the scale of the ‘urban’ was minute and did not produce sociosexual and iatrogenic conditions that would favor viral amplification, as was the case in Kinshasa or Douala [5]. Therefore, the designation of Bissau as a ‘major urban area’ is a contemporary representation, not a historical fact. This raises questions about the inferences underlying the perfect storm narrative: the exceptionalism of the urban, the rural-to-capital population movements, explosive urban growth marked by a sex-ratio imbalance, ‘anomic’ urban behaviors (namely, sex work), STD epidemics and amplification via iatrogenic routes involving en masse campaigns, namely, against human African trypanosomiasis (HAT).

Neither the colony as a whole nor the present-day capital experienced major developments during the colonial period. Unlike other contexts, the lack of a thriving industry meant that the urban ‘beacon’ did not attract the general population. The ethnographic data reinforce these ideas, illustrating that mobility patterns were not Bissau-driven. Instead, rural-to-rural migration patterns fed on old commercial and kinship networks that crisscrossed the country and the Senegambia region for centuries. Throughout the colonial period, people walked the land, as no regular mechanical or riverine transport industry existed [47]. In Bissau, neither the idea of scores of people practicing commercial sex, nor the colonial discourse of rampant sexual diseases, materialized. Archival health data counter the narratives of STD epidemics in Bissau and elsewhere, namely, throughout the reputed epidemic years, (1955–1970) [23,46,48]. Ultimately, these data question the exceptional nature attributed to urban areas, while redirecting the focus to rural Guinea-Bissau as the unlikely, yet extraordinary, location for epidemic ignition. The indirect effects of the war on ecological relations, mobility and sociability were acute in the rural community and are key to the HIV-2 epidemic.

### 3.2. A Transforming War 

Thucydides’ account of the Athenian plague of 435 BC established the relationship between war and epidemics [49]. Therefore, unsurprisingly, war was smoothly integrated into the perfect storm model as a trait of the HIV-2 epidemic, despite the fact that no research had ever detailed how it might actually have contributed. The present research identified an indirect link between the conflict and the amplification of the epidemic. In 1963, the PAIGC started a decade-long war of liberation. Two years later, the war had taken a toll on the colonial regime with a loss of 50% of the territory and c. 33% (160,000) of the population [50]. By 1968, the geographical loss rose to 75%. Thousands of individuals in areas newly liberated by the PAIGC were forced to leave their villages and move into the bush in the southern and northern regions, or neighboring countries. They built makeshift villages, planted agricultural patches, collected food and/or hunted to survive [51,52]. This meant new and stressful ecological human/primate relations, with plenty of opportunities for the viral interspecies jump to occur. This spillover might result from manipulation for consumption [53,54]—the cut-hunter idea—but also from other activities, such as pet-keeping [55], the competition for food forest resources, crop protection [21,54,56] and/or traditional medicinal purposes [54,57].

Even in the midst of a war, Africans did not remain in villages. They continued to move on foot across conflict areas and international borders throughout the conflict. Portuguese military intelligence noted 13,000 individuals, 3000 of them foreigners (Senegalese, Guineans and Gambians), coming into Guinea-Bissau on a monthly basis to visit relatives and engage in commerce or use health facilities [58,59]. This meant that war led to an average of 120,000 individuals regularly moving in/out of the country—critical information when considering the regional spread of the virus.

The liberation war proved seismic. To combat ideas of independence, the Portuguese re-militarized the health services, integrating biomedicine into counter-guerrilla programs (Acção psico-social—PSYOPS—psycho-social action), thus contributing to a wartime expansion of the services. This was accompanied by a public health approach that further favored injection procedures. The systematic use of injections in rural areas related to consultations, military psyops, multi-disease actions and epidemic responses. Therefore, the mimicking of the perfect storm model is thus troublesome in this context. 

Portugal used healthcare to try to win hearts and minds and counter the trend towards independence. This translated into a re-militarization and expansion of the health services in the hinterland and border areas. In the Portuguese colonies, as elsewhere under colonialism, health was a political tool [22,60]. The PAIGC created a prototype health system in order to affirm its statehood status [51,52]. However, this was a makeshift service, due to the scarce human and material resources and a focus on war-related injuries. Portuguese attacks confined permanent structures (hospitals and other facilities) to the Republic of Guinea and to Senegal. Ultimately, this service was incapable of triggering and supporting the epidemic’s spread.

### 3.3. Public Health Politics, Biotechnology Uses and the Early Spread of HIV-2A 

Throughout the 20th century, the Portuguese colonial health service favored invasive biomedical practices to care for the population. The reshuffling of the service following the onset of the liberation/colonial war emphasized this. This path to parenteral transmission interacted above ecological and social transformations.

Biomedical technology acted as a reliable bridge between simians and humans. The infection of two individuals with HIV-2 in the 1960s [61,62], after receiving blood transfusions in Bissau, demonstrates the relative certainty that the virus was passed by this means. However, the small scale of transfusions at the height of the war—1872 in 1971 [63] to 6177 in 1973 [58]—and their centralization in the capital indicate a secondary role in the spread of the epidemic. 

**Figure 2:**
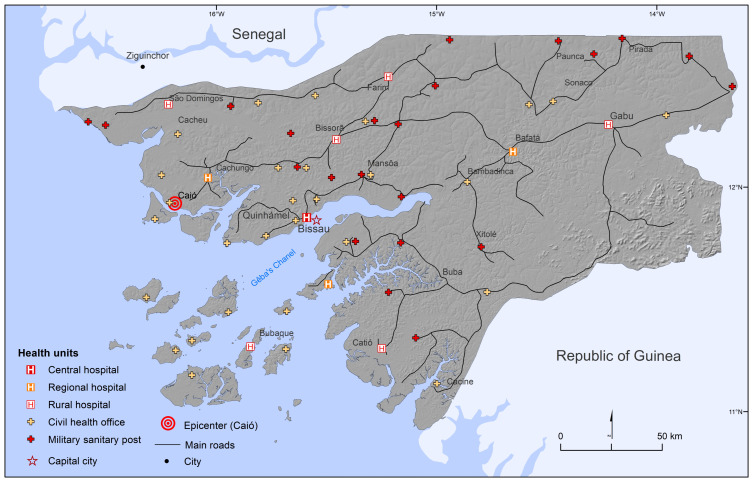
Map of roads and government health facilities, 1972. Source: Adapted by Author from Guinea-Bissau 1972. [64].

After the end of the pacification wars in the 1930s, the state began to establish small health facilities in the hinterland [23], geographically centered in the west and north of the country (Figure 2). In the 1950s, mobile HAT campaigns helped cover the hinterland. Injection procedures accompanied the expansion of the health services there throughout these decades. Notwithstanding the twelvefold growth in the number of injections and vaccinations between 1930 and 1950 [23], roughly two-thirds of the population was still not inoculated. Thus, even if the virus had already adapted to humans (tMRCA 1928–1947), the amplification had yet to occur: notably, it is the exponential surge in injections during the liberation/colonial war that reveals when it did occur (Table 2). During this period, the ratio of injections jumped ninefold, from 0.3 in the 1930s–1950s to 2.8 per year per person, which is almost double the world average in 1998 [31]. The uses of injections must be considered the main source of iatrogenic transmission in Guinea-Bissau. 

Importantly, this increase in injections was not without its flaws. As elsewhere in Africa, an economy of scarcity molded daily biomedical care operations on the ground [5,6]. In the early 1960s, the anti-TB program aimed to immunize the whole population, estimated at 500,000. Remarkably, the blueprint report of the TB service says it received only 166 glass syringes and 4130 needles for this task [70]. This material was distributed to the 74 health facilities and four mobile teams (Table 3). As detailed below, sterilizing material was also scarce and inappropriate, while staff were overburdened with work, namely, at the small permanent facilities. This context was conducive to iatrogenic transmission. 

However, closer attention to data reveals that there were several opportunities for the occurrence of amplification under the open skies of rural Guinea-Bissau. Historical research allows us to detail the daily working conditions of different biomedical practices—consultations, military programs (PSYOPS), multi-disease actions and epidemic responses —and evaluate how they may have contributed to viral transmission. From the serial passage perspective, these practices made viral amplification possible due to the structurally impromptu conditions that undermined the provision of safe injections.

Consultations often included using injections for treatment and/or vaccination, and biomedical acts could be conducted in permanent health facilities, military barracks, PSYOPs programs or en masse campaigns. The military PSYOPs teams visited villages, and examined, treated or gave injections to individuals, regardless of the politics or military situation [50]. By 1972, PSYOPS surpassed all the other services, permanent and mobile, in the number of consultations provided [59]. En masse campaigns, which usually focused on colonial-controlled areas, became less regular as the war progressed. Each concentração (gathering), most of which were held under military supervision for security reasons, tackled a wide range of ailments and included epidemic responses. In December 1964 and January 1965, the government responded to a yellow fever outbreak scare with a mass vaccination campaign that reached 200,000 people—an astonishing rate of 3333 injections/day. As in other parts of colonial Africa, this employed syringes with glass barrels and reusable needles and rudimentary kerosene stoves and boiling pans for sterilization [71], which meant there was plenty of room for iatrogenic transmission. This took place in the hinterland and frontier regions of the north, east and south of the country, namely, the Farim (north), Gabú (east) and Catío (south) areas, where some parts were ‘liberated’, but where Portugal still had military garrisons [66]. 

**Figure 3:**
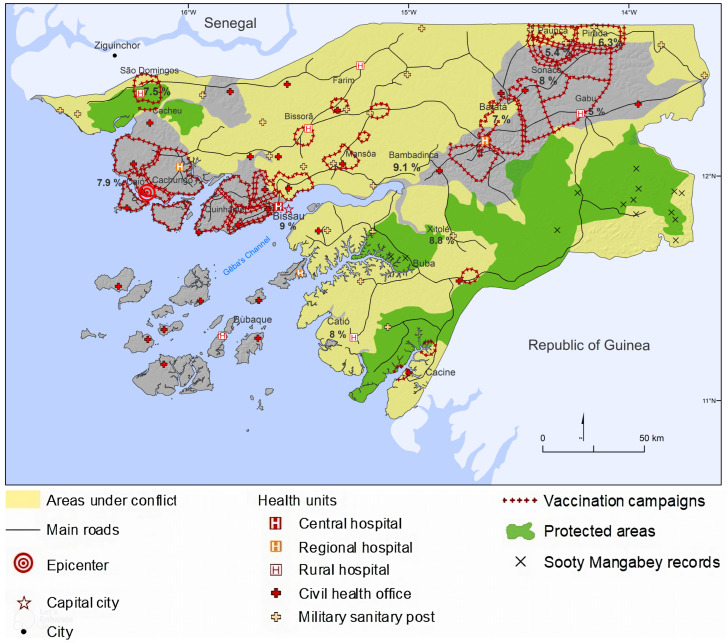
Map: Not the perfect storm- of overlapping colonial areas, medical health service units and mass injection and vaccination campaigns, percentage of HIV-2 prevalence and location of sooty mangabeys. Source: original map by authors adapted from [28,51,64,66,74].

Figure 3 reveals that these actions took place in areas within the natural range of sooty mangabeys and thus included people who were possibly exposed to SIVsm. Hundreds of thousands of inoculations (preventive or therapeutic) were carried out in consultations, en masse actions, outreach military psychosocial programs or epidemic responses. They targeted a now smaller population pool located in restricted colonial areas or the vicinities of health and/or military structures that remained active in liberated areas. A closer look at operational details and geographical data counters the idea that the anti-human African trypanosomiasis (mission to fight trypanosomiasis, MCT [missões de combate às tripanosomiases]) and TB [4,72] mobile campaigns were key factors in the epidemic spread. In the case of Guinea-Bissau, the war had curtailed these programs to areas surrounding clinics or army barracks while also integrating them into larger multi-disease endeavors. Men, women and children were assembled by the local administration for treatment or vaccination in both colonial-controlled areas and liberated zones where there were still government garrisons. These public-health events were the perfect opportunity to tackle a range of diseases and train future health auxiliaries in injection techniques [73]. However, the services continually struggled with a lack of personnel and material resources, such as glass syringe barrels, needles and sterilization materials, thus leading to widespread unsafe injection practices. 

### 3.4. Common Viral Trails (HCV and HTLV-1) 

More information on the likely iatrogenic spread of HIV-2 can also be found by examining the cases of other viruses in the territory. Attention to other viruses, such as human T-cell lymphotropic/leukemia/lymphoma virus type 1 (HTLV-1) and hepatitis C virus [HCV] genotype 2, provides valuable information about past iatrogenic transmission. Even if these are not benign diseases, they are good markers, due to their biological characteristics, asymptomatic nature and their low mortality and strong cohort effect [53,72,75,76,77]. 

Interestingly, in addition to HIV-2, the village of Caió had the highest prevalence of HTLV-1 in West Africa—roughly 6%, or double that of Bissau [78]. Another attribute is the strong age cohort effect among elderly individuals. HTLV-1 followed the decrease in the prevalence of HIV-2 from 1990 to 2007, although it has recently slightly increased (2016), solely among children, thus emphasizing vertical rather than iatrogenic transmission factors (i.e., breastfeeding) [79].

The characteristics of an HCV epidemic—an unusually high prevalence, a strong cohort effect and a steady decline over time—are parallel to those of HIV-2 [80,81]. Recent studies have found that individuals aged 50 years or over in Guinea-Bissau were particularly affected by HCV, which suggests historical exposures via unsterile medical procedures. Millennials, on the other hand, present no signs of infection [76,81]. Unfortunately, the lack of geographically detailed studies (on Caió, for instance) makes it difficult to prove the direct link between unsafe injections and viral spread, of the kind that has been demonstrated in Egypt or Cameroon [72,77,82,83,84,85]. Taken together, these studies suggest the importance of iatrogenic transmission during colonial public health programs as the key driver of the HIV-2 epidemic. 

The high prevalence of HIV-2 found in individuals with vaccinia scars from smallpox vaccination, particularly those from Caió [79], underlines a process of iatrogenic transmission during either consultations, inoculation actions or epidemic responses promoted by colonial services. Its proximity to Bissau and the fact that it was home to 42 percent of the population mean that the west of the country (an area that included the county of Cachungo and the supposed epicenter, the village of Caió) received particular attention. This new analysis of the data helps explain how a tiny village in a tropical cul-de-sac region came to be named the epicenter of HIV-2. After independence, these conditions were not present on the same scale.

### 3.5. Post-Independence 

The perfect storm model does not explain the high prevalence of HIV-2 in towns and villages, rather than in the larger cities in countries bordering Guinea-Bissau. The first epidemiological studies on HIV in West Africa revealed the pervasive dissemination of HIV-2 in the Guinea-Bissauan, Senegalese and Gambian hinterlands [86,87,88,89]. These studies exemplified the unorthodox uses of injection technology, for instance, “nurses and pharmacists often treat [ed] genital ulcers quite inappropriately with a 10-day course of penicillin” [12] (Pépin et al., 1991:73). The high prevalence of unsterile injections in this region suggests that parenteral transmission was a key pathway for the spread of the HIV virus until the 21st century [90]. In Africa, blood transfusions are intrinsically a family/household affair [91]—a rationale that hints at the existence of networks of biotechnology use, such as injections. Attention to the social use of these technologies, namely, by kinship networks, thus helps explain the identical viral sequences found in towns/cities or remote locations like Caió [76,92].

## 4. Discussion

The perfect storm model for HIV-1M fails to explain how HIV epidemics come about, namely, in the case of HIV-2. It fails to explain the location, timing and path of this specific West-African epidemic and its spillover. In Guinea-Bissau, it is the rural milieu that presents all the relevant factors, making it the ‘ignitor’ of the HIV-2 epidemic. This setting had the natural host of the virus, the population numbers, the mobility trends and the use of technology on a scale needed to foster viral adaptation and amplification. Careful analysis of the transformations of ecological relations, mobility and sociability offers plausible explanations for the epidemic spread of an ill-adapted virus. However, it was the indirect impact of the war via biomedical practices that brought together humans, monkeys and the virus in new ways under the open skies of makeshift villages or in the vicinity of rural clinics. Several public health circumstances—consultations, vaccination campaigns, military operations and epidemic responses—may inevitably have contributed to the adaptation of a simian virus to the human organism and its epidemic spread. 

The colonial/liberation war was more than a politically transforming period, in that it also gave rise to other sociopolitical changes and healthcare practices that favored viral adaptation and amplification. It triggered changes in mobility, with populations escaping to forests or neighboring countries. The new makeshift villages and gardens led to increased proximity to primates in the forests. Individuals continued to move within/to colonial or liberated areas and/or neighboring countries. The continuous interventions of the health services in rural areas during the conflict encountered a population exposed to SIVsm and helped to significantly expand transmission on a massive scale—an initial step in a broader amplification process. Overlapping all this was the intensive use of biomedical technology that acted as a bridge to transfer a new virus. The combination of an expansion of healthcare practices throughout the hinterland and border areas and a re-emphasizing of invasive medical practices such as inoculations (injections and vaccination) gained in scale, despite the scarce material and human resources and a ‘restless’ population pool. Populations were subjected to intense use of injection technology under inadequate conditions. Our findings further underline that the location of viral emergence must be reconsidered. The existence of sooty mangabeys in Guinea-Bissau and the high prevalence of the HIV-2 epidemic hint at a more localized spillover, rather than an event across thousands of kilometers following travel by a single infected simian/human. 

Our data and analysis reveal that the rural location was the real ‘igniter’ in Guinea-Bissau. In the case of HIV-2, this was a country in conflict, which presented socio–eco–political elements that helped shape viral biology into adaptation and epidemic spread. A correlation between HIV-2 emergence and parenteral transmission can be identified during the colonial period for HCV and HTLV-1. However, more precise studies with phylogeographic data are needed.

## 5. Conclusions

In the context of the present study, historical data provide detailed information on the HIV virus during the 20th century; in particular, emphasis is given to the last decade of the colonial period and a new narrative suggests profound differences from the perfect storm model in the emergence of this virus. Special attention was paid to the war’s indirect influence on viral adaptation and spread. Many activities could also have generated the spillover from simians to humans in Guinea-Bissau. The colonial/liberation war intensified human mobility and exposure to invasive medical practices, facilitating the adaptation of a simian virus to human transmission. The present study has shown that the quality and safety of biomedical care in areas where humans and primates are in contact is paramount in avoiding iatrogenic transmissions. Caution is thus essential when using the one-size-fits-all model for viral emergences. Historical data may not allow us to say when, where and how this HIV, or other viruses for that matter, surfaced, but it can impose caution in relation to flawed narratives of perfect storms. Empirically based investigations bring other drivers of new disease emergence into a clearer focus.

## Figures and Tables

**Table 1:** The urban and rural populations of Guinea-Bissau, 1940s–1970s.

	1940	1950	1960	1970	1979
Total population	345,267	508,970	521,336	487,448	767,731
Bissau	1898	17,321	25,524	64,242	105,273
Total % of rural pop.	98.9%	95.3%	94%	87%	84.5%

Source: Adapted from [46].

**Table 2:** Injections and vaccinations in Guinea-Bissau, 1930s–1970s.

Year	Number of Injections and Vaccinations
1933	22,029
1935	13,758
1940	42,199
1945	102,704
1950	63,415
1955	160,650
1957	77,207
1960	292,049
1965	473,135
1970	503,467

Sources: Adapted by author from [23,45,65,66,67,68,69].

**Table 3:** Distribution of injection materials to the anti-TB service (1962).

Number (No.) of Syringes and Needles per TB Health Facility (1962)			
Health-Facilities	Number of Facilities	No. Syringes Per Facility	No. Needles Per Facility
BGC Center	1		
BGC County Centers	10	4	200
BCG Vaccination Post	64	2	30
Mobile Teams	4	6	750
Total number		166	4130

Source: Adapted by author from [70].

## Data Availability

Not applicable.
